# Subjective socioeconomic status moderates the relationship between objective neighborhood disadvantage and quality of life in middle- and older-aged women with breast cancer

**DOI:** 10.1007/s10549-026-08008-1

**Published:** 2026-06-26

**Authors:** Rachel L. Plotke, Millan R. Kanaya, Paula J. Popok, Sarah N. Webster, Jenna L. Hansen, Mason J. Krueger, Mary Roberts, Sara Ebrahimi, Estefany Saez-Clarke, Chloe J. Taub, Neha Goel, Michael H. Antoni

**Affiliations:** 1 Department of Psychology, University of Miami, 5665 Ponce de Leon Blvd, Floor 5, Coral Gables, FL 33146 USA; 2 Division of General Internal Medicine and Population Science, Department of Medicine, University of Alabama at Birmingham Heersink School of Medicine, Birmingham, AL USA; 3 UAB O’Neal Comprehensive Cancer Center, Cancer Control and Population Science Program, Birmingham, AL USA; 4 Breast Service, Department of Surgery, Memorial Sloan Kettering Cancer Center, New York, NY USA; 5 Sylvester Comprehensive Cancer Center, University of Miami, Miami, FL USA

**Keywords:** Breast cancer, Neighborhood disadvantage, Area deprivation index, Subjective socioeconomic status, Perceived social standing, Quality of life, Health disparities

## Abstract

**Purpose:**

Living in a disadvantaged neighborhood is linked to higher mortality rates and poorer quality of life (QoL) among patients with breast cancer (BC). Subjective socioeconomic status (SSS), reflecting one’s perceived socioeconomic “rank” or social standing relative to others, may be associated with differences in the relationship between objective neighborhood disadvantage and QoL. Therefore, we sought to evaluate whether SSS moderated the association between objective neighborhood disadvantage and QoL in middle- and older-aged women undergoing BC treatment.

**Methods:**

Women (*≥* 50yrs) diagnosed with non-metastatic BC participating in a stress management trial completed a baseline assessment of SSS (MacArthur Network Sociodemographic Questionnaire) and QoL (Functional Assessment of Cancer Therapy-Breast) in the weeks after surgery. The Area Deprivation Index (ADI), which ranks the degree of neighborhood disadvantage via participants’ addresses, measured objective neighborhood disadvantage. Multivariate linear regressions related ADI, SSS, and QoL, adjusting for age, cancer stage, and race/ethnicity.

**Results:**

Greater SSS relative to the community (*B* = 3.60, *SE* = 1.12, *p*=.002) and the USA (*B* = 3.01, *SE* = 1.09, *p*=.006) related to better QoL. Also, SSS USA interacted with ADI in predicting QoL (*B* = 0.95, *SE* = 0.45, *p*=.038), such that greater ADI related to poorer QoL in women with lower but not higher SSS.

**Conclusion:**

Greater SSS relative to one’s community and the USA population related to better QoL in women treated for BC. Conversely, greater objective neighborhood disadvantage related to poorer QoL but not among those with greater SSS. Future work could examine whether SSS is a modifiable intervention target through coping effectiveness training, or by enhancing community and social engagement.

**Supplementary Information:**

The online version contains supplementary material available at 10.1007/s10549-026-08008-1.

## Background

Socioeconomic factors such as low income and poor housing quality are well-documented in their negative impact on breast cancer disease trajectory and outcomes [1, 2], despite significant improvements in treatment methods in the past decades. One’s built and social environment, or neighborhood, are known to be key determinants of health [3–5]. Neighborhood disadvantage, an objective measure of neighborhood-level socio-environmental disparities (e.g., poverty, unemployment), has been linked to physical and mental health disparities including higher breast cancer mortality rates, more aggressive tumor biology [1, 2] and lower quality of life (QoL) [6] compared to individuals living in advantaged neighborhoods. These disparities may in part be explained by the cumulative toll of adverse environments on chronic stress [7]. However, one’s perceived social standing, otherwise referred to as subjective socioeconomic status (SSS), may moderate the association between objective socioeconomic adversity and overall QoL in breast cancer patients undergoing treatment.

Although associations between objective socioeconomic status (SES) and QoL are established in cancer and non-cancer populations alike [8–13], subjective socioeconomic status may better reflect individual differences in one’s daily lived experience or “reality” as they move through challenging periods. Unlike traditional indicators of objective SES, subjective socioeconomic status represents a person’s perception of their own place within a social hierarchy relative to others [14, 15] and is described as a more nuanced measure of social status and predictor of health [16]. Conceptualizing subjective socioeconomic status within a social hierarchy in this way can reflect a number of factors including respectability, recognition by others, social influence, power, and control [17]. Numerous studies have demonstrated that poorer perceived social status is a key indicator of negative health outcomes [13, 16, 18], irrespective of objective SES indicators [14, 19–22]. Thus, while both objective SES and subjective socioeconomic status are independent indicators of health outcomes including QoL, few studies have examined the interacting effects of these variables in the context of breast cancer treatment. Moreover, such work has not examined interactions between subjective socioeconomic status and an objective neighborhood-level index, such as the Area Deprivation Index (ADI), to represent neighborhood-level SES in a sample of breast cancer patients, despite well-established links between ADI and psychological adversity measures in women with breast cancer [7].

The present study explores associations between objective neighborhood disadvantage (i.e., ADI), subjective socioeconomic status, and QoL among mid-to-older aged women with non-metastatic breast cancer in the post-surgical period. More specifically, we investigated the moderating effect of subjective socioeconomic status— comparing oneself to the USA population and to the local community— on the relationship between ADI and QoL. We hypothesized that better subjective socioeconomic status (i.e., higher SSS community and higher SSS USA) would be associated with better QoL, and greater neighborhood disadvantage (i.e., higher ADI) would be associated with poorer QoL overall. We also hypothesized that subjective socioeconomic status would moderate the relationship between neighborhood disadvantage and QoL, such that higher ADI would be related to poorer QoL only among those also reporting worse subjective socioeconomic status (i.e., lower SSS community and SSS USA).

## Methods

### Study eligibility and procedures

Participants were recruited from oncology clinics in Southern Florida between July 2016 and May 2023 to participate in a stress management intervention as part of a larger trial (ClinicalTrials.gov ID: NCT03955991), approved by the University of Miami Institutional Review Board (#20160525). Eligible participants were women with newly diagnosed stage 0-III breast cancer, who had completed primary breast cancer surgery but had not yet begun adjuvant therapy, and were ≥ 50 years of age. Further inclusion criteria were 6th -grade English reading level, life expectancy > 12 months, and at least moderate distress, as determined by scores *≥* 14 on the Impact of Event Scale-Intrusion subscale [23] or self-reported moderate distress over the past week using a single item (scoring *≥* 4 on a scale of 1–10). Exclusion criteria were a history of cancer (except for non-melanoma skin cancer) within the past two years, major psychopathology (e.g., schizophrenia, psychosis), an active diagnosis of major depressive disorder, panic disorder, post-traumatic stress disorder, suicidal ideation, or substance dependence in the past 12-months, comorbid medical conditions affecting the immune system (e.g., HIV, autoimmune conditions), current immunomodulator medication, and cognitive impairment, as determined by scores *≤* 31 on the Telephone Interview for Cognitive Status [24]. Following screening, 109 eligible patients enrolled in the study and provided informed consent.

### Study design

The current study is a cross-sectional, secondary analysis of baseline data in the setting of a remotely-delivered cognitive behavioral stress management (R-CBSM) intervention trial. Participants completed baseline measures, including a self-report psychosocial assessment battery, before being randomized into a group-based R-CBSM intervention or waitlist control group. This study utilizes data from participants who completed the study variables of interest at baseline and had full address information for determining ADI (*N* = 99).

### Measures

#### Primary outcome variable

##### Quality of life

Quality of life (QoL) was measured using the Functional Assessment of Cancer Therapy-Breast (FACT-B) [25]. This 37-item scale assesses four general domains of QoL (i.e., Physical Well-Being [PWB], Social/Family Well-Being [SFWB], Emotional Well-Being [EWB], Functional Well-Being [FWB]) as well as Breast Cancer Subscale (BCS). Each item was self-rated on a 5-point Likert scale ranging from “Not at all” to “Very much,” where participants were asked to indicate their response as it applied to the past 7 days. Some items were reverse coded to derive subscale and total scores, with higher subscale and total scores indicating better QoL. The FACT-B total score ranges from 0 to 148, PWB, SFWB, and FWB subscales range from 0 to 28, EWB subscale from 0 to 24, and BCS from 0 to 40. The FACT-B total score demonstrated excellent internal consistency in our sample (α = 0.92). The subscales demonstrated the following internal consistencies: PWB (α = 0.85), SFWB (α = 0.83), FWB (α = 0.85), EWB (α = 0.76), BCS (α = 0.65).

#### Moderator variable

##### Subjective socioeconomic status

Subjective socioeconomic status (SSS) was measured using the MacArthur Network Sociodemographic Questionnaire [14]. Participants self-reported an “X” on a 10-rung “social ladder” to indicate their perceived social standing, relative to others in their community (i.e., SSS community) and others in the United States (i.e., SSS USA). Higher rungs indicate higher subjective socioeconomic status. There are several advantages to using this type of subjective rank-based measure. Scholars interpret its mechanisms as reflecting two distinct concepts: perceived non-economic social status and perceived economic circumstances [17]. Others emphasize how it uniquely captures individuals’ self-evaluations of their social standing through perceptions of resources, and where those resources place them relative to others via social-comparative processes, making it distinct from objective SES measures [26]. This conceptualization of subjective socioeconomic status allows for the assessment of multiple domains that can be understood as a “cognitive average” of non-economic social status and objective SES indicators, derived through individuals’ cognitive appraisals. Overall, this measure is widely accepted and utilized and shows appropriate test-retest reliability [27].

##### *Subjective socioeconomic status – Community*

To assess SSS community, participants were shown an image of a ladder with the following prompt: “Think of this ladder as representing where people stand in their communities. People define community in different ways; please define it in whatever way is most meaningful to you. At the top of the ladder are people who have the highest standing in their community. At the bottom are the people who have the lowest standing in their community. Please indicate where you think you stand at this time in your life, relative to other people in your community. Where would you place yourself on this ladder?”

##### *Subjective socioeconomic status – United States*

To assess SSS USA, participants were shown an image of a ladder with the following prompt: “Think of this ladder as representing where people stand in the United States. At the top of the ladder are the people who are the best off - those who have the most money, the most education, and the most respected jobs. At the bottom are the people who are the worst off - those who have the least money, least education, the least respected jobs, or no job. The higher up you are on this ladder, the closer you are to the people at the very top; the lower you are, the closer you are to the people at the very bottom. Please indicate where you think you stand at this time in your life, relative to other people in the United States. Where would you place yourself on this ladder?”

#### Predictor variable

##### Objective neighborhood disadvantage

Objective neighborhood disadvantage was measured using the Area Deprivation Index (ADI) [28]. The ADI is a validated composite index that captures 17 measures across five domains of neighborhood disadvantage including income (e.g., income disparity), employment (e.g., percentage of civilian labor force population aged *≥* 16 years unemployed), education (e.g., percentage of population aged *≥* 25 years with at least a high school diploma), housing (e.g., median home value), and household characteristics (e.g., percentage of single-parent households with children aged *≤* 18 years) [26]. The ADI was calculated by linking participants’ addresses to the census block group level via the ADI mapping atlas (https://www.neighborhoodatlas.medicine.wisc.edu/mapping). ADI scores expressed decile values based on state-level statistics and ranged from 1 to 10, with higher scores indicating greater neighborhood disadvantage.

#### Covariates

Age, breast cancer stage (0-III), and race/ethnicity (coded Hispanic, non-Hispanic White, non-Hispanic Black, and other) were included and selected a priori based on prior literature relevant to the current study to account for potential bias [7, 29], with covariate inclusion limited by the small sample size. Covariates were verified using the participant’s electronic medical record.

### Statistical analyses

Analyses were conducted using the Statistical Package for the Social Sciences (SPSS) Version 29. Data were screened for normality and outliers. Participants with missing data for main outcomes were excluded from analyses. For models including SSS, listwise deletion was used to handle missing data. Descriptive analyses were used to summarize participant characteristics. Linear regression models assessed direct effects between SSS and ADI on QoL, as well as the moderating effect of subjective socioeconomic status on the relationship between ADI and QoL. Moderation analyses were conducted using SPSS PROCESS Macro [30]. Significant moderation analyses were probed using simple slopes at low (-1SD), mean, and high (+1SD) levels of the moderator. Effect size for tests of moderation, *f*^*2*^, was used, where *f*^*2*^ *≥* 0.005 represents a small effect size, *f*^*2*^ *≥* 0.01 represents a medium effect size, and *f*^*2*^ *≥* 0.025 represents a large effect size [31]. Post-hoc analyses were not corrected for multiple comparisons due to being exploratory in nature.

## Results

### Participant characteristics

The sample consisted of 99 participants with a mean age of 60.7 years (*SD* = 7.56). The majority of women identified as Non-Hispanic White (41%), followed by Hispanic (36%). Most women had stage I breast cancer (60%) and underwent a lumpectomy (57%). Mean ADI was 3.08 (*SD* = 2.50) and mean FACT-B total score was 104.90 (*SD* = 22.23) (Table 1). Mean SSS community (i.e., perceived social standing relative to others in their community) was 6.88 (*SD* = 1.95) and mean SSS USA (i.e., perceived social standing relative to others in the USA) was 6.68 (*SD* = 2.12). There were no significant differences in SSS ratings or ADI values by Hispanic ethnicity. There were no significant differences in women reporting low (-1SD), average (Mean), and high (+ 1SD) SSS community, SSS USA, or ADI for days since surgery, surgery type, disease stage or grade, age, or comorbidities (Charlson Comorbidity Index) [32].

**Table 1:** Demographic and clinical characteristics, and study variables

Variable	*N* = 99
Age at Enrollment (years), *M (SD)*	60.78 (7.56)
Race / Ethnicity, *N (%)*	
Hispanic	36 (36.4)
Non-Hispanic White	41 (41.4)
Non-Hispanic Black	13 (13.1)
Other / No response	9 (9.1)
Education (years), *M (SD)*	15.3 (2.86)
Marital Status, *N (%)*	
Married / Partnered	53 (53.5)
Divorced / Separated	29 (29.3)
Widowed	8 (8.1)
Single, Never Married	9 (9.1)
Stage at Diagnosis, *N (%)*	
0	13 (13.8)
I	56 (59.6)
II	21 (22.3)
III	4 (4.3)
Surgery Type, *N (%)*	
Lumpectomy	54 (56.8)
Mastectomy	41 (43.2)
Time since surgery at baseline (days), *M (SD)*	61.13 (64.16)
SSS Community: Perceived social standing relative to others in your community, *M (SD)*	6.88 (1.95), range 2–10
SSS USA: Perceived social standing relative to others in the USA, *M (SD)*	6.68 (2.12), range 2–10
ADI (state), *M (SD)*	3.08 (2.50), range 1–10
FACT-B total score, *M (SD)*	104.90 (22.23)
FACT-B PWB	21.82 (5.70)
FACT-B SFWB	21.70 (5.75)
FACT-B EWB	18.08 (4.21)
FACT-B FWB	17.66 (6.44)
FACT-B BCS	26.96 (6.30)

Note. SSS = Subjective Socioeconomic Status; ADI = Area Deprivation Index; FACT-B = Functional Assessment of Cancer Therapy-Breast; PWB = Physical Well-Being; SFWB = Social/Family Well-Being; EWB = Emotional Well-Being; FWB = Functional Well-Being; BCS = Breast Cancer Subscale

Additional descriptive clinical and demographic characteristics and study variables are described in Table 1, and sociodemographic characteristics from the MacArthur Sociodemographic Questionnaire are described in Table 2.

**Table 2:** The MacArthur sociodemographic questionnaire

Variable	*N* = 99
Highest grade completed, *N (%)*	
High school or less (grades 1–12)	25 (26.9)
College (grades 13–16)	40 (43.0)
Graduate or more (17–20+)	28 (30.1)
Highest degree earned, *N (%)*	
High school diploma or equivalent (GED)	14 (14.9)
Associate degree	23 (24.5)
Bachelor’s degree	30 (31.9)
Master’s degree	18 (19.1)
Doctorate or Professional (MD, JD, DDS, etc.)	9 (9.6)
Working status, *N (%)*	
Full-time or part-time	52 (58.4)
Unemployed / Laid off / Looking for work /	11 (12.3)
Disability	
Keeping house or raising children	3 (3.4)
Retired	23 (25.8)
Personal income last 12 months, *N (%)*	
$0 – $34,999	18 (18.2)
$35,000 – $99,999	36 (36.4)
* ≥*$100,000	15 (15.1)
No response	30 (30.3)
People living in household, *N (%)*	
One	21 (23.9)
Two	31 (35.2)
Three	25 (28.4)
Four or more	11 (12.5)
Adults in household, *N (%)*	
One	31 (34.1)
Two	37 (40.7)
Three or more	23 (25.3)
Children in household, *N (%)*	
None	67 (74.4)
One	13 (14.4)
Two or more	10 (11.1)
Adults in household with income, *N (%)*	
None	7 (8.1)
One	37 (43.0)
Two	32 (37.2)
Three or more	10 (11.7)
Combined household income last 12 months, *N (%)*	
$5,000 – $34,999	12 (12.1)
$35,000 – $99,999	28 (28.3)
* ≥*$100,000	29 (29.3)
No response	30 (30.3)
Home type, *N (%)*	
Owned	71 (77.2)
Rented	19 (20.7)
Other	2 (2.2)
Amount of time able to live at current address and standard of living if all sources of household income were lost, *N (%)*	
Less than 1 month	6 (9.0)
1 to 2 months	11 (16.4)
3 to 6 months	6 (9.0)
7 to 12 months	10 (14.9)
More than 1 year	34 (50.7)
Financial assets, *N (%)*	
<$4,999	11 (11.1)
$5,000 – $19,999	7 (7.0)
$20,000 – $99,999	6 (6.0)
$100,000 – $499,999	11 (11.1)
>$500,000	15 (15.2)
Don’t know / No response	49 (49.5)
Net worth, *N (%)*	
<$4,999	16 (16.2)
$5,000 – $19,999	4 (4.0)
$20,000 – $99,999	6 (6.0)
$100,000 – $499,999	9 (9.1)
>$500,000	14 (14.1)
Don’t know / No response	50 (50.5)

### Main effect multivariate models

The complete results of main effect multivariate models can be found in Table 3. After adjusting for covariates, greater SSS community (*B* = 3.60, *SE* = 1.12, *p* =.002) and greater SSS USA (*B* = 3.07, *SE* = 1.09, *p* =.006) significantly related to better overall QoL. ADI was not significantly related to overall QoL (*B* = -0.83, *SE* = 0.94, *p* =.380).

**Table 3:** Multivariate regression models with area deprivation index, subjective socioeconomic status, and quality of life total score and subscales

Outcome	Predictor	B	SE	Standardized β	*p*	Total Model *R*^2^	F, *p*-value
FACT-B Total Score	ADI	-0.83	0.94	-0.09	0.380	0.058	*F*[4,89] = 1.37, *p*=.252
FACT-B Total Score	SSS Community	3.60	1.12	0.33	0.002**	0.162	*F*[4,83] = 4.01, *p*=.005**
FACT-B PWB	SSS Community	0.72	0.28	0.27	0.011*	0.127	*F*[4,83] = 3.03, *p*=.022*
FACT-B SFWB	SSS Community	0.38	0.28	0.15	0.183	0.029	*F*[4,83] = 0.62, *p*=.648
FACT-B EWB	SSS Community	0.58	0.22	0.27	0.012*	0.107	*F*[4,83] = 2.49, *p*=.049*
FACT-B FWB	SSS Community	1.01	0.32	0.31	0.002**	0.169	*F*[4,83] = 4.22, *p*=.004**
FACT-B BCS	SSS Community	0.85	0.33	0.26	0.012*	0.124	*F*[4,83] = 2.92, *p*=.026*
FACT-B Total Score	SSS USA	3.07	1.09	0.29	0.006**	0.136	*F*[4,84] = 3.32, *p*=.014*
FACT-B PWB	SSS USA	0.84	0.26	0.33	0.002**	0.155	*F*[4,84] = 3.84, *p*=.006**
FACT-B SFWB	SSS USA	0.49	0.27	0.20	0.070	0.048	*F*[4,84] = 1.06, *p*=.384
FACT-B EWB	SSS USA	0.47	0.22	0.23	0.035*	0.089	*F*[4,84] = 2.05, *p*=.095
FACT-B FWB	SSS USA	0.78	0.31	0.25	0.015*	0.135	*F*[4,84] = 3.26, *p*=.015*
FACT-B BCS	SSS USA	0.49	0.33	0.16	0.140	0.074	*F*[4,84] = 1.69, *p*=.160

Note. All models control for age, cancer stage, and race/ethnicity

SSS = Subjective Socioeconomic Status; ADI = Area Deprivation Index; FACT-B = Functional Assessment of Cancer Therapy-Breast; PWB = Physical Well-Being; SFWB = Social/Family Well-Being; EWB = Emotional Well-Being; FWB = Functional Well-Being; BCS = Breast Cancer Subscale

*p <.05

**p <.01

***p <.001

### Interaction effects of ADI and SSS on quality of life

**Fig. 1:**
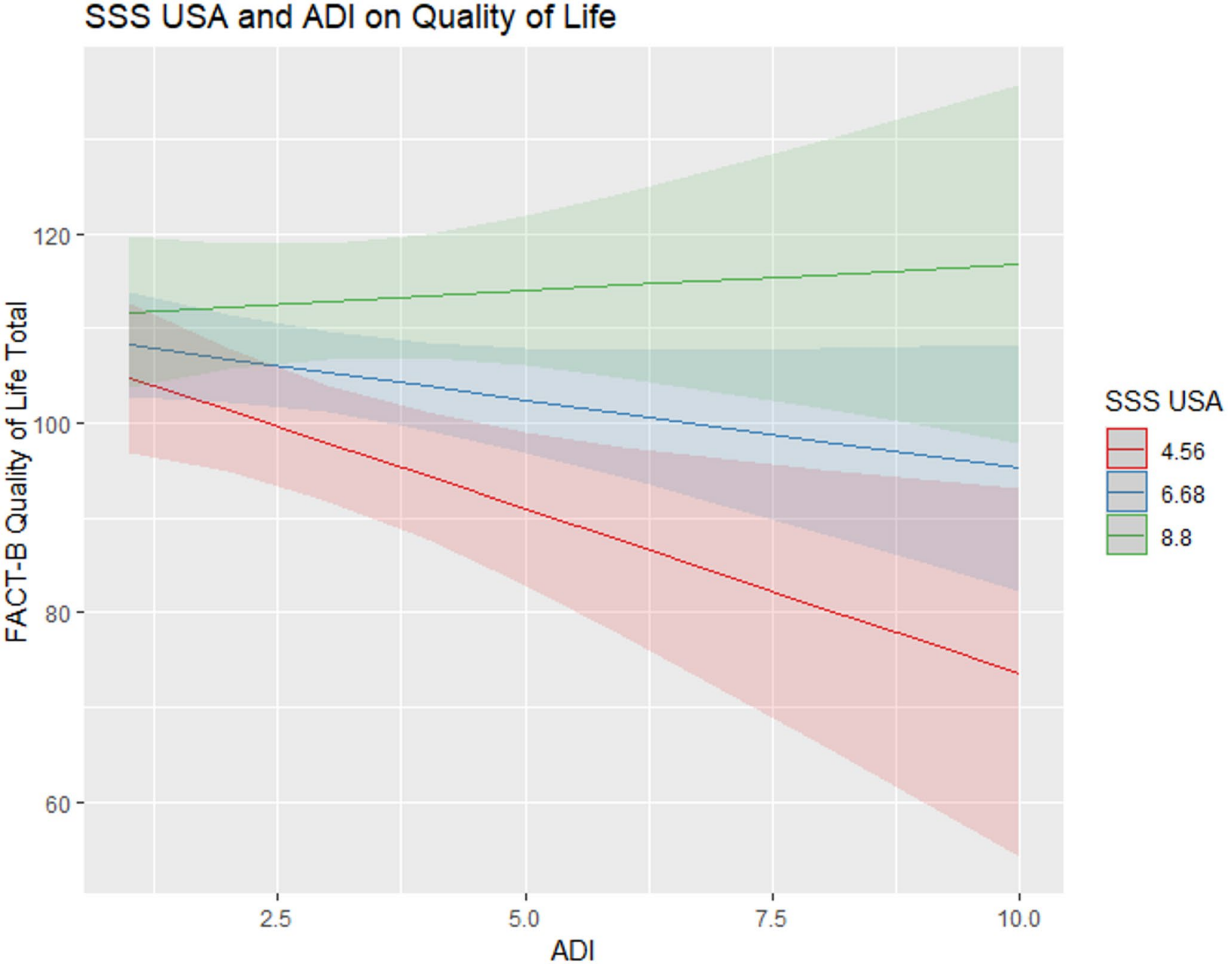
Interaction effects of subjective socioeconomic status USA on the relationship between area deprivation index and overall quality of life

SSS USA significantly moderated the relationship between ADI and QoL (*B* = 0.95, *SE* = 0.45, *p* =.038), with a large effect size (*f*^*2*^ = 0.05). The overall model was significant (*R*^2^ = 0.181, *F* [4,89] = 4.91, *p* <.01; Table 4). Simple slope analysis revealed that the interaction effect of ADI on QoL was significant at low levels of SSS USA (-1SD = 4.56 *p* =.009), but not at middle (M = 6.68, *p* =.103) or high levels of SSS USA (+ 1SD = 8.80 *p* =.664; Fig. 1), such that higher ADI (i.e., greater neighborhood disadvantage) related to poorer QoL among women with low subjective socioeconomic status relative to others in the USA. SSS community did not significantly moderate the relationship between ADI and QoL (*p* >.05).

**Table 4:** Interaction effects of area deprivation index and subjective socioeconomic status USA on overall quality of life

FACT-B Quality of Life Total Score				
Variable	Unstandardized B	SE	95% CI	*p*
Constant	106.05	12.17	[81.87, 130.23]	< 0.001***
ADI	-7.80	3.15	[-14.04, -1.54]	0.015*
SSS USA	0.68	1.68	[-2.67, 4.03]	0.688
ADI x SSS USA	0.95	0.45	[0.06, 1.84]	0.038*

Total Model *R*^*2*^ = 0.181, *F* [4, 89] = 4.91, *p* <.01**

Note. ADI = Area Deprivation Index; SSS = Subjective Socioeconomic Status

*p <.05

**p <.01

***p <.001

### Exploratory post-hoc analyses

Given the significant main effect models examining SSS community and SSS USA on overall QoL, exploratory post-hoc analyses were conducted in order to examine the relationship between SSS and the FACT-B subscales.

#### Main effects of SSS on quality of life subscales

Greater SSS community related to better Physical Well-Being (*B* = 0.72, *SE* = 0.28, *p* =.011), Emotional Well-Being (*B* = 0.58, *SE* = 0.22, *p* =.011), Functional Well-Being (*B* = 1.01, *SE* = 0.32, *p* =.002), and Breast Cancer Subscale (*B* = 0.85, *SE* = 0.32, *p* =.012). Similarly, greater SSS USA related to better Physical Well-Being (*B* = 0.84, *SE* = 0.26, *p* =.002), Emotional Well-Being (*B* = 0.47, *SE* = 0.22, *p* =.035), and Functional Well-Being (*B* = 0.78, *SE* = 0.31, *p* =.015). SSS USA was not significantly associated with Breast Cancer Subscale (*B* = 0.49, *SE* = 0.33, *p* =.140), and SSS community and SSS USA were not significantly associated with Social/Family Well-Being (*B* = 0.38, *SE* = 0.28, *p* =.183; *B* = 0.49, *SE* = 0.27, *p* =.070; Table 3).

#### Interaction effects of ADI and SSS on quality of life subscales

**Fig. 2:**
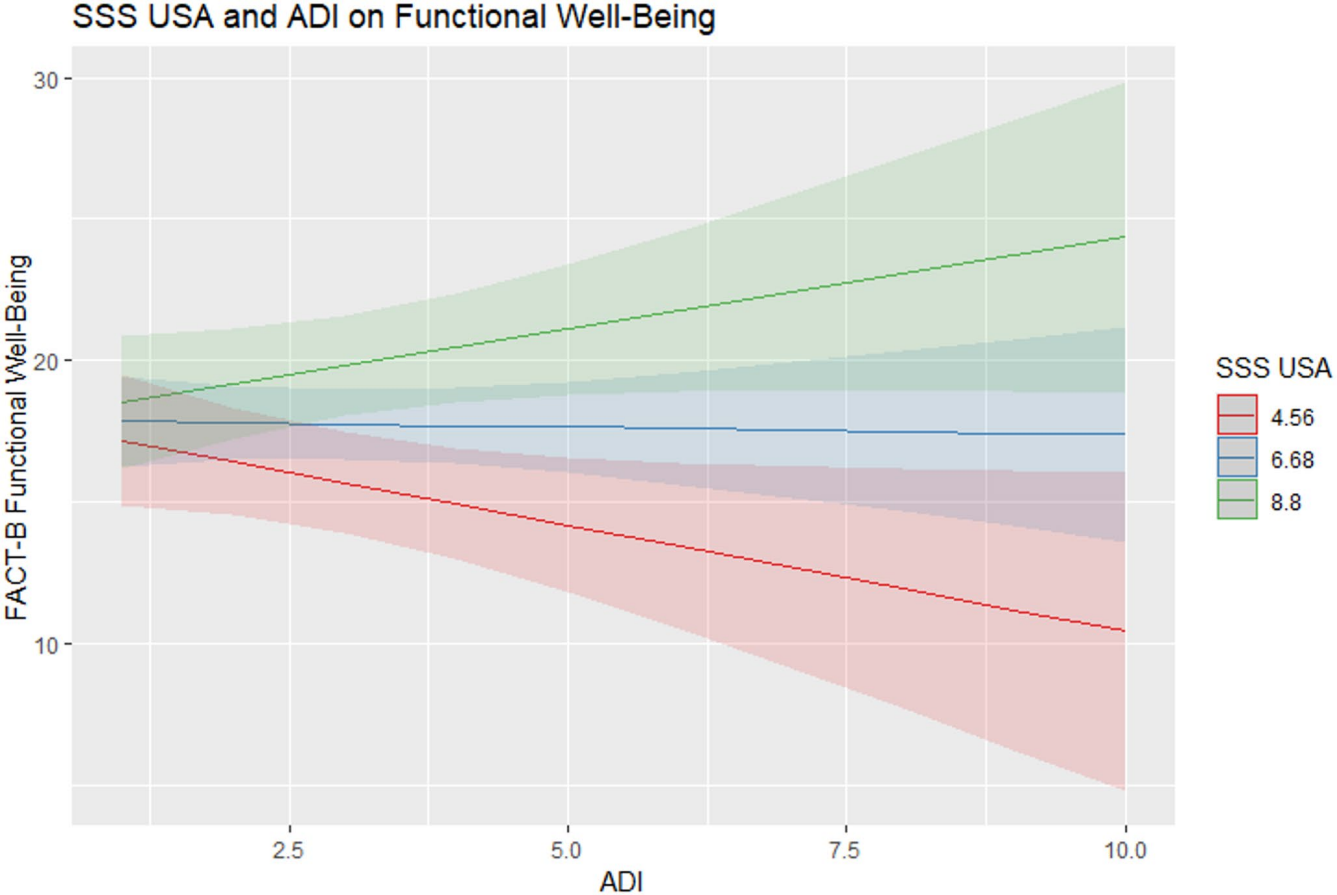
Interaction effects of subjective socioeconomic status USA on the relationship between area deprivation index and functional well-being

SSS USA significantly moderated the relationship between ADI and Functional Well-Being (*B* = 0.33, *SE* = 0.13, *p* =.014), with the overall model being significant (*R*^2^ = 0.171, *F* [4,89] = 4.60, *p* <.01; Table 5). Simple slope analysis revealed such that the interaction effect of ADI on Functional Well-Being was significant at low levels of SSS USA (-1SD = 4.56 *p* =.044), but not at middle (M = 6.68, *p* =.737) or high levels of SSS USA (+ 1SD = 8.80 *p* =.106; Fig. 2), such that higher ADI related to poorer Functional Well-Being among women with low subjective socioeconomic status relative to others in the USA. Although SSS community significantly moderated the relationship between ADI and Functional Well-Being (*B* = 0.29, *SE* = 0.14, *p* =.040), simple slope analyses did not reveal statistical significance at any level of SSS community. No other moderation models were statistically significant (Supplementary Tables 1–10).

**Table 5:** Interaction effects of area deprivation index and subjective socioeconomic status USA on functional well-being

FACT-B Functional Well-Being Subscale				
Variable	Unstandardized B	SE	95% CI	*p*
Constant	18.52	3.54	[11.49, 25.56]	< 0.001***
ADI	-2.25	0.92	[-4.07, -0.43]	0.016*
SSS USA	-0.01	0.49	[-0.99, 0.96]	0.979
ADI x SSS USA	0.33	0.13	[0.07, 0.59]	0.014*

Total Model *R*^*2*^ = 0.171, *F* [4, 89] = 4.60, *p* <.01**

Note. ADI = Area Deprivation Index; SSS = Subjective Socioeconomic Status

*p <.05

**p <.01

***p <.001

## Discussion

The current study investigated associations between objective neighborhood disadvantage, subjective socioeconomic status, and QoL in a diverse sample of middle-aged and older women recently diagnosed with breast cancer. Greater subjective socioeconomic status (i.e., SSS community and SSS USA) related to better QoL overall. Exploratory post-hoc analyses examining the QoL subscales further revealed that greater subjective socioeconomic status related to better physical well-being, emotional well-being, and functional well-being for SSS community and SSS USA, as well as better breast cancer-specific QoL for SSS community. Although objective neighborhood disadvantage was not associated with overall QoL across the entire sample, results demonstrated an interaction effect of ADI x SSS on QoL, which indicated that greater neighborhood disadvantage related to lower QoL only among individuals reporting lower subjective socioeconomic status relative to others in the USA. Furthermore, exploratory post-hoc analyses revealed a conditional effect of ADI on functional well-being indicating that greater neighborhood disadvantage related to lower functional well-being only among individuals reporting lower subjective socioeconomic status relative to others in the USA.

Our findings are consistent with prior literature demonstrating that subjective social status is an important determinant of health outcomes [13, 14, 16, 18–22]. This association is well-established but is predominantly limited to noncancer populations examining physical and psychological health, rather than breast cancer-specific QoL. For example, a meta-analysis conducted by Cundiff and colleagues [33] overwhelmingly revealed that subjective socioeconomic status was associated with self-rated and biomarker indicators of physical health, as well as specific symptoms and disease diagnoses, independent of objective neighborhood-level SES. Other research conducted among a sample of healthy middle-aged women found associations between SSS USA and both self-reported psychological measures and health behaviors [19]. Specifically, lower SSS community significantly related to greater levels of anxiety, pessimism, and stress, while lower SSS USA significantly related to poorer healthy dietary and exercise behaviors [19]. Finally, in one study conducted with patients following hematopoietic stem cell transplantation, researchers found that higher ratings of subjective socioeconomic status related to less depressive symptoms and generalized distress, fewer symptoms of post-traumatic stress disorder, and better QoL [13].

In our second aim, we investigated the association between objective neighborhood disadvantage and QoL. Contrary to some research, neighborhood disadvantage did not independently relate to overall QoL in our sample. However, this finding does align with a growing body of evidence suggesting that subjective socioeconomic status, rather than objective indicators of SES like income, education, and neighborhood structural elements, shows stronger associations with psychological well-being and self-rated health [14, 20, 34]. This is not to suggest that neighborhood disadvantage or other objective SES factors are insignificant– the way in which we form perceptions are often shaped by objective characteristics. For instance, in a recent study conducted by Taub and colleagues [7], greater objective neighborhood disadvantage was associated with higher perceived neighborhood disadvantage in a cohort of patients with breast cancer. Here, perceived neighborhood disadvantage was measured by the Neighborhood Social Environment Adversity Survey which assesses multiple domains of the neighborhood environment, including safety, social cohesion, aesthetic quality, and walkability [35, 36]. In contrast, the present study assessed subjective social standing within a broader social hierarchy that extends beyond neighborhood-specific conditions. Nevertheless, the link between subjective socioeconomic status and QoL and psychological health may operate through some elements of objective disadvantage. Thus, an individual’s appraisals of neighborhood factors and broader perceptions of social status may be more closely linked to health outcomes.

Several theories may help contextualize these findings. For instance, the relativity hypothesis highlights the role of social comparative processes in relation to SES [26]. Applied to the present study, the relativity hypothesis proposes that individuals evaluate their own social standing relative to others in shaping their QoL, rather than by absolute SES factors. There is well-established literature demonstrating that engaging in social comparisons can affect QoL among cancer patients. For example, Bouchard and colleagues [37] examined this process in women recently diagnosed with nonmetastatic breast cancer. They found that more frequent upward contrast social comparisons (i.e., considering oneself as being worse off than women without breast cancer) and downward identification social comparisons (i.e., considering oneself as being similar to women whose breast cancer is worse than theirs and imagining the possibility of being in their situation) were associated with poorer QoL in the post-surgical period. Although this work focused on breast cancer-specific comparisons rather than SES, the underlying function of social comparison processes is relevant for understanding the impact on QoL. Other researchers argue that subjective socioeconomic status may be more strongly related to health over traditional SES markers as explained by the cognitive averaging principle [38]. This principle posits that subjective socioeconomic status represents a “cognitive average” of objective SES indicators (e.g., occupation, financial status, education) drawn from individuals’ appraisals of their social standing and thus a more precise assessment of SES [16, 20, 34, 38]. Given that SES is complex and multifaceted, its effects on health may be better understood through subjective evaluations, as subjective socioeconomic status may function as a composite of both subjective and objective SES.

Our findings further suggest that self-perceptions of social standing are associated with differences in how objective neighborhood disadvantage relates to QoL in women undergoing treatment for breast cancer, as illustrated in the moderation analysis. Despite both SSS USA and SSS community significantly relating to QoL, interestingly, results revealed that only SSS USA significantly moderated the relationship between ADI and overall QoL, which was an unexpected finding. These findings raise an interesting question about the relative importance of community versus national perceptions of social status. One potential explanation for these observed differences between SSS community and SSS USA may be related to differences in frame of reference. For instance, comparisons relative to one’s community may be more proximal, familiar, or socially similar, such that these shared circumstances may outweigh objective neighborhood disadvantage factors. Conversely, the SSS USA ladder prompts broader, more distal comparisons to the national population, which may magnify perceptions of inequality and create a more evident sense of disconnect.

Moreover, the MacArthur ladder questions use different language to assess subjective socioeconomic status. Unlike the community ladder, the USA ladder explicitly references SES indicators including money, education, and occupation as markers of subjective social standing, suggesting that perceptions grounded in specific SES factors may carry greater significance. Accounting for these nuanced differences in interpretation provides further evidence that subjective socioeconomic status is not a substitute for objective SES; rather, it is inherently implicated with it and underscores the need to consider subjective evaluations of SES as it relates to health. Given this differential pattern between SSS USA and SSS Community, future research should aim to replicate and further examine this exploratory observation.

Finally, among the FACT-B QoL subscales, only functional well-being emerged as significant in the exploratory post-hoc moderation analysis, suggesting that functional well-being was compromised in women reporting lower SSS USA and living in a more disadvantaged neighborhood. The functional well-being subscale specifically assesses one’s ability to engage in meaningful activities such as work and work at home, feeling fulfillment from these activities, as well as overall contentment and enjoyment in life [27]. Essentially, this subscale reflects women’s daily functioning capabilities while undergoing breast cancer treatment.

Functional well-being is regarded as an essential component of QoL, which may explain its unique emergence as the only significant subscale in exploratory post-hoc analyses. Many women experience functional limitations following a breast cancer diagnosis, considerably hindering their abilities to engage in activities of daily living at the same level prior to their diagnosis and treatment [39, 40]. For example, Michael and colleagues [41] found that mid- to older-aged women with breast cancer reported significantly greater functional decline over time compared to their non-cancer counterparts, as evidenced by reduced physical, role, and social function, lower energy and vitality, as well as increased pain. As it relates to the current study, women living in more disadvantaged neighborhoods with poorer subjective social status may face additional barriers resulting in exacerbated functional limitations. For example, if occupational status is a salient basis for social comparisons, being unable to work may contribute to poorer self-perceptions. Furthermore, disadvantaged neighborhoods are inherently associated with greater social adversity [1, 7] in addition to poorer functional well-being among older adults [10]. Thus, neighborhood-level adversity may exacerbate functional limitations due to poor walkability and public transportation infrastructure, safety concerns, or lack of social resources [10], and having poorer self-perceptions may further hinder abilities to seek out or receive support. Taken together, these factors may significantly diminish functional well-being among women undergoing treatment for breast cancer.

### Strengths and limitations

This study has several strengths worth noting. The study sample was ethnically diverse, particularly with respect to Hispanic populations, and included a wide range of breast cancer diagnoses. Further, this study utilized the ADI, which incorporates 17 indicators of objective neighborhood-level SES across five domains of neighborhood disadvantage, providing a more holistic assessment than individual SES metrics commonly used in prior research. This study ultimately provides novel findings that can guide future intervention targets, as the interaction between ADI, subjective socioeconomic status, and QoL had not been previously examined.

Despite its strengths, this study also has notable limitations to consider. The cross-sectional design restricts causal inferences and limits longitudinal insights. The relatively small sample size may have reduced statistical power and limited our ability to detect clinically significant effects, while the characteristics of the sample may have limited generalizability. Participants included in analyses were predominantly highly educated, skewed towards lower levels of neighborhood disadvantage, and were required to meet a minimum distress threshold as part of the stress management intervention trial. The distress-based inclusion criterion is important to consider, particularly in the context of interpreting QoL results, as it likely resulted in a sample with elevated distress and corresponding lower QoL relative to the broader breast cancer population. Furthermore, these sample characteristics may help explain the lack of observed associations between ADI and QoL.

An additional limitation relates to the interpretation of effect size for the moderation analysis. The *f*^*2*^ benchmarks [31] were developed for categorical moderators, and caution is warranted when applying them to a continuous moderator such as SSS USA in the current study. Additionally, a sensitivity power analysis conducted using G*Power [42] indicated that our study was powered to detect a minimum interaction effect of approximately *f*^*2*^ *=* 0.09. Findings should therefore be interpreted with these considerations in mind. Future research would benefit from building on this work using a larger, more socioeconomically diverse sample, and one that is more representative of the general breast cancer population.

### Clinical implications and future research

Findings from the current study highlight the importance of the interaction between subjective socioeconomic status and objective neighborhood disadvantage on QoL, underscoring the clinical implications in patients with breast cancer. Patients living in disadvantaged neighborhoods who also perceive themselves as lower in social standing may be more vulnerable to poorer QoL, underscoring the need for targeted psychosocial support for this group. Interventions that address maladaptive appraisals related to social comparisons and perceived social status may be particularly beneficial, alongside enhancing overall self-efficacy and coping skills. For example, using a technique called Coping Effectiveness Training (CET) [43, 44], which helps patients differentiate between controllable and uncontrollable socio-environmental stressors and apply appropriate coping strategies, may reduce the detrimental effects of living in disadvantaged neighborhoods. Cognitive behavioral approaches, such as cognitive behavioral stress management (CBSM) and acceptance-based strategies, encompass CET and other coping skills training, and are plausible intervention approaches for this population.

Furthermore, it is beneficial for researchers and clinicians alike to consider the implications of broader social determinants of health (e.g., social support, community cohesion) rather than relying solely on objective neighborhood-level SES metrics. Incorporating self-report measures of subjective social experiences into oncology settings may help clinicians identify “at-risk” patients in order to make appropriate referrals to psychosocial services. From a research perspective, future studies should explore the underlying drivers of subjective socioeconomic status that could be modifiable intervention targets, as well as examine whether subjective socioeconomic status is associated with attenuation of negative health outcomes of neighborhood disadvantage, such as neuroendocrine stress markers, disease progression, or long-term survival.

## Conclusions

Findings from the current study showed that greater subjective socioeconomic status related to better QoL, but that living in a disadvantaged neighborhood alongside lower subjective socioeconomic status related to compromised QoL in women dealing with the challenges of treatment for breast cancer. This study builds on evidence that higher subjective social standing is linked with better health outcomes and contributes novel findings by demonstrating that subjective socioeconomic status impacts the relationship between objective neighborhood disadvantage and QoL in women undergoing treatment for breast cancer. Our study provides a foundation for future research to investigate mechanisms underlying the observed associations between subjective socioeconomic status and QoL in order to tailor psychosocial interventions to improve QoL, especially among individuals living in more disadvantaged neighborhoods.

## Supplementary Information

Below is the link to the electronic supplementary material.

Supplementary Material 1

## Data Availability

The datasets generated during and/or analyzed during the current study are available from the corresponding author on reasonable request.
