# Supplementary material for: Subjective socioeconomic status moderates the relationship between objective neighborhood disadvantage and quality of life in middle- and older-aged women with breast cancer
Source: Breast Cancer Res Treat. 2026 Jun 26;217(3):61. doi: 10.1007/s10549-026-08008-1 (PMC13309438; doi:10.1007/s10549-026-08008-1)
Supplement: Supplementary file 1 — Supplementary Material 1 [file 10549_2026_8008_MOESM1_ESM.docx]

| **Supplementary Table 1.**  *Interaction Effects of Area Deprivation Index and Subjective Socioeconomic Status Community on Overall Quality of Life* | | | | |
| --- | --- | --- | --- | --- |
| **FACT-B Quality of Life Total Score** | | | | |
| **Variable** | ***Unstandardized B*** | ***SE*** | **95% CI** | ***p*** |
| Constant | 97.36 | 13.11 | [71.32, 123.41] | **<.001***** |
| ADI | -5.78 | 3.23 | [-12.20, 0.63] | .077 |
| SSS Community | 1.76 | 1.82 | [-1.86, 5.37] | .337 |
| ADI x SSS Community | 0.70 | 0.47 | [-0.23, 1.63] | .140 |
| Total Model *R^2^* = .166, *F* [4, 88] = 4.37, *p* < .01** | | | | |
| *Note*. ADI = Area Deprivation Index; SSS = Subjective Socioeconomic Status  **p* < .05.  ***p* < .01.  ****p* < .001. | | | | |

| **Supplementary Table 2.**  *Interaction Effects of Area Deprivation Index and Subjective Socioeconomic Status Community on Physical Well-Being* | | | | |
| --- | --- | --- | --- | --- |
| **FACT-B Physical Well-Being Subscale** | | | | |
| **Variable** | ***Unstandardized B*** | ***SE*** | **95% CI** | ***p*** |
| Constant | 19.82 | 3.45 | [12.96, 26.67] | **<.001***** |
| ADI | -1.19 | 0.85 | [-2.88, 0.50] | .165 |
| SSS Community | 0.39 | 0.48 | [-0.56, 1.34] | .415 |
| ADI x SSS Community | 0.14 | 0.12 | [-0.10, 0.39] | .254 |
| Total Model *R^2^* = .108, *F* [4, 88] = 2.65, *p* < .05* | | | | |
| *Note*. ADI = Area Deprivation Index; SSS = Subjective Socioeconomic Status  **p* < .05.  ***p* < .01.  ****p* < .001. | | | | |

| **Supplementary Table 3.**  *Interaction Effects of Area Deprivation Index and Subjective Socioeconomic Status Community on Social/Family Well-Being* | | | | |
| --- | --- | --- | --- | --- |
| **FACT-B Social/Family Well-Being Subscale** | | | | |
| **Variable** | ***Unstandardized B*** | ***SE*** | **95% CI** | ***p*** |
| Constant | 20.56 | 3.29 | [14.02, 27.11] | **<.001***** |
| ADI | -0.71 | 0.81 | [-2.32, 0.90] | .383 |
| SSS Community | 0.38 | 0.46 | [-0.53, 1.28] | .414 |
| ADI x SSS Community | 0.05 | 0.12 | [-0.18, 0.29] | .641 |
| Total Model *R^2^* = .076, *F* [4, 88] = 1.81, *p* > .05 | | | | |
| *Note*. ADI = Area Deprivation Index; SSS = Subjective Socioeconomic Status  **p* < .05.  ***p* < .01.  ****p* < .001. | | | | |

| **Supplementary Table 4.**  *Interaction Effects of Area Deprivation Index and Subjective Socioeconomic Status Community on Emotional Well-Being* | | | | |
| --- | --- | --- | --- | --- |
| **FACT-B Emotional Well-Being Subscale** | | | | |
| **Variable** | ***Unstandardized B*** | ***SE*** | **95% CI** | ***p*** |
| Constant | 15.38 | 2.57 | [10.27, 20.49] | **<.001***** |
| ADI | -0.52 | 0.63 | [-1.78, 0.74] | .417 |
| SSS Community | 0.43 | 0.36 | [-0.28, 1.14] | .234 |
| ADI x SSS Community | 0.06 | 0.09 | [-0.12, 0.24] | .525 |
| Total Model *R^2^* = .091, *F* [4, 88] = 2.19, *p* > .05 | | | | |
| *Note*. ADI = Area Deprivation Index; SSS = Subjective Socioeconomic Status  **p* < .05.  ***p* < .01.  ****p* < .001. | | | | |

| **Supplementary Table 5.**  *Interaction Effects of Area Deprivation Index and Subjective Socioeconomic Status Community on Functional Well-Being* | | | | |
| --- | --- | --- | --- | --- |
| **FACT-B Functional Well-Being Subscale** | | | | |
| **Variable** | ***Unstandardized B*** | ***SE*** | **95% CI** | ***p*** |
| Constant | 17.08 | 3.83 | [9.46, 24.70] | **<.001***** |
| ADI | -1.88 | 0.94 | [-3.76, 0.00] | .050 |
| SSS Community | 0.17 | 0.53 | [-0.89, 1.23] | .751 |
| ADI x SSS Community | 0.29 | 0.14 | [0.01, 0.56] | **.040*** |
| Total Model *R^2^* = .156, *F* [4, 88] = 4.06, *p* < .01** | | | | |
| *Note*. ADI = Area Deprivation Index; SSS = Subjective Socioeconomic Status  **p* < .05.  ***p* < .01.  ****p* < .001. | | | | |

| **Supplementary Table 6.**  *Interaction Effects of Area Deprivation Index and Subjective Socioeconomic Status Community on Breast Cancer Subscale* | | | | |
| --- | --- | --- | --- | --- |
| **FACT-B Breast Cancer Subscale** | | | | |
| **Variable** | ***Unstandardized B*** | ***SE*** | **95% CI** | ***p*** |
| Constant | 24.43 | 3.74 | [17.00, 31.87] | **<.001***** |
| ADI | -1.10 | 0.92 | [-2.93, 0.74] | .238 |
| SSS Community | 0.52 | 0.52 | [-0.52, 1.55] | .322 |
| ADI x SSS Community | 0.10 | 0.13 | [-0.17, 0.36] | .464 |
| Total Model *R^2^* = .107, *F* [4, 88] = 2.63, *p* < .05* | | | | |
| *Note*. ADI = Area Deprivation Index; SSS = Subjective Socioeconomic Status  **p* < .05.  ***p* < .01.  ****p* < .001. | | | | |

| **Supplementary Table 7.**  *Interaction Effects of Area Deprivation Index and Subjective Socioeconomic Status USA on Physical Well-Being* | | | | |
| --- | --- | --- | --- | --- |
| **FACT-B Physical Well-Being Subscale** | | | | |
| **Variable** | ***Unstandardized B*** | ***SE*** | **95% CI** | ***p*** |
| Constant | 20.47 | 3.12 | [14.27, 26.67] | **<.001***** |
| ADI | -1.67 | 0.81 | [-3.28, -0.07] | **.041*** |
| SSS USA | 0.32 | 0.43 | [-0.53, 1.18] | .455 |
| ADI x SSS USA | 0.20 | 0.12 | [-0.03, 0.43] | .081 |
| Total Model *R^2^* = .161, *F* [4, 89] = 4.25, *p* < .01* | | | | |
| *Note*. ADI = Area Deprivation Index; SSS = Subjective Socioeconomic Status  **p* < .05.  ***p* < .01.  ****p* < .001. | | | | |

| **Supplementary Table 8.**  *Interaction Effects of Area Deprivation Index and Subjective Socioeconomic Status USA on Social/Family Well-Being* | | | | |
| --- | --- | --- | --- | --- |
| **FACT-B Social/Family Well-Being Subscale** | | | | |
| **Variable** | ***Unstandardized B*** | ***SE*** | **95% CI** | ***p*** |
| Constant | 21.46 | 3.03 | [15.45, 27.47] | **<.001***** |
| ADI | -1.19 | 0.78 | [-2.74, 0.37] | .134 |
| SSS USA | 0.26 | 0.42 | [-0.57, 1.10] | .530 |
| ADI x SSS USA | 0.12 | 0.11 | [-0.10, 0.34] | .292 |
| Total Model *R^2^* = .106, *F* [4, 89] = 2.63, *p* < .05* | | | | |
| *Note*. ADI = Area Deprivation Index; SSS = Subjective Socioeconomic Status  **p* < .05.  ***p* < .01.  ****p* < .001. | | | | |

| **Supplementary Table 9.**  *Interaction Effects of Area Deprivation Index and Subjective Socioeconomic Status USA on Emotional Well-Being* | | | | |
| --- | --- | --- | --- | --- |
| **FACT-B Emotional Well-Being Subscale** | | | | |
| **Variable** | ***Unstandardized B*** | ***SE*** | **95% CI** | ***p*** |
| Constant | 17.07 | 2.42 | [12.26, 21.89] | **<.001***** |
| ADI | -0.83 | 0.63 | [-2.07, 0.42] | .190 |
| SSS USA | 0.22 | 0.34 | [-0.45, 0.88] | .518 |
| ADI x SSS USA | 0.10 | 0.09 | [-0.08, 0.28] | .279 |
| Total Model *R^2^* = .085, *F* [4, 89] = 2.06, *p* > .05 | | | | |
| *Note*. ADI = Area Deprivation Index; SSS = Subjective Socioeconomic Status  **p* < .05.  ***p* < .01.  ****p* < .001. | | | | |

| **Supplementary Table 10.**  *Interaction Effects of Area Deprivation Index and Subjective Socioeconomic Status USA on Breast Cancer Subscale* | | | | |
| --- | --- | --- | --- | --- |
| **FACT-B Breast Cancer Subscale** | | | | |
| **Variable** | ***Unstandardized B*** | ***SE*** | **95% CI** | ***p*** |
| Constant | 27.09 | 3.61 | [19.91, 34.27] | **<.001***** |
| ADI | -1.21 | 0.93 | [-3.06, 0.65] | .200 |
| SSS USA | 0.20 | 0.50 | [-0.79, 1.20] | .684 |
| ADI x SSS USA | 0.10 | 0.13 | [-0.16, 0.37] | .441 |
| Total Model *R^2^* = .075, *F* [4, 89] = 1.81, *p* > .05 | | | | |
| *Note*. ADI = Area Deprivation Index; SSS = Subjective Socioeconomic Status  **p* < .05.  ***p* < .01.  ****p* < .001. | | | | |
